# Front-line of genome editing technology for animal cell engineering

**DOI:** 10.1186/1753-6561-9-S9-O1

**Published:** 2015-12-14

**Authors:** Tetsushi Sakuma

**Affiliations:** 1 Department of Mathematical and Life Sciences, Graduate School of Science, Hiroshima University, 1-3-1 Kagamiyama, Higashi-Hiroshima, Hiroshima 739-8526, Japan

## Background

Genome editing technology heralds a new era for animal cell engineering. Programmable site-specific nucleases, such as transcription activator-like effector nucleases (TALENs) and clustered regularly-interspaced short palindromic repeats (CRISPR)/Cas9, enable to induce DNA double-strand breaks (DSBs) at any desired genomic loci, resulting in efficient gene knockout and knock-in in broad range of cultured cells [1].

As for gene knock-in, homologous recombination (HR)-assisted method has generally been used for spontaneous or programmable nuclease-mediated donor DNA integration. It enables precise gene knock-in, but the labor for constructing targeting vector with long homology arms and limited applicability due to the lower HR activity have been technical hurdles to utilize this method.

## Materials and methods

Our group has so far developed various systems in genome editing field, such as the Platinum Gate TALEN system for constructing highly-active Platinum TALENs [2,3] and the Multiplex CRISPR/Cas9 Assembly System for creating all-in-one CRISPR/Cas9 vector enabling highly-efficient multiplex genome editing in cells and animals [4,5]. Recently, along with the use of these systems, our group newly established the PITCh (Precise Integration into Target Chromosome) system, which facilitates convenient gene knock-in in cultured cells and organisms [6,7].

## Results and conclusions

Our PITCh system utilizes an alternative DSB repair pathway, microhomology-mediated end-joining (MMEJ), which enables easy, efficient and precise gene knock-in without relying on HR. Using the PITCh system, we have successfully shown gene knock-in in human cells, silkworms, zebrafish, and frogs [6,7]. Importantly, there had been no report of successful gene knock-in in frogs because of low HR frequency.

**Figure 1:**
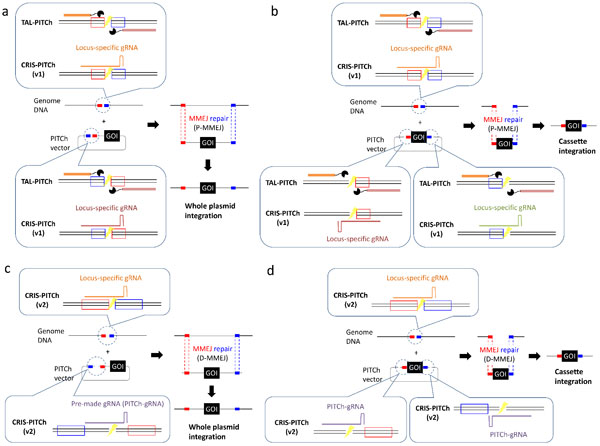
**The PITCh systems for gene knock-in mediated by MMEJ**. The original PITCh systems, TAL-PITCh and CRIS-PITCh (v1), utilize proximal MMEJ (P-MMEJ) (a, b), whereas the modified PITCh system, CRIS-PITCh (v2), utilizes distal MMEJ (D-MMEJ) (c, d). PITCh systems enable both whole plasmid integration (a, c) and particular cassette integration without carrying the plasmid backbone sequence (b, d). Red and blue boxes indicate the microhomologous sequence.

Along with some modifications after the publication of the original paper, the PITCh system now enables backbone-free, direction-oriented, and non-mutagenic integration of large gene cassette in various cells and organisms with simple construction of the unique donor vector, termed PITCh vector (Figure 1).We believe that this method will provide technical and practical contributions to a wide range of researchers interested in TALEN- or CRISPR/Cas9-based gene knock-in in a variety of cells and organisms.
